# Supplementary material for: End Sequence Analysis Toolkit (ESAT) expands the extractable information from single-cell RNA-seq data
Source: Genome Res. 2016 Oct;26(10):1397–410. doi: 10.1101/gr.207902.116 (PMC5052061; doi:10.1101/gr.207902.116)
Supplement: Supplemental Material [file supp_gr.207902.116_Supp_Materials.tar.gz › ESAT-esat_v0.1/doc/html/dir_7e6fcd4ad5f319a381b9e40bdf47c42a.html]

ESAT: java/broad/core/math Directory Reference


ESAT


- Main Page
- Related Pages
- Packages
- Classes
- Files

All Classes Namespaces Files Functions Variables Enumerator Pages

- java
- broad
- core
- math

math Directory Reference

Directory dependency graph for math:

|  |  |
| --- | --- |
| Files | |
| file | ANOVA.java |
|  | |
| file | CombinationGenerator.java |
|  | |
| file | ComputeFDR.java |
|  | |
| file | Distribution.java |
|  | |
| file | EmpiricalDistribution.java |
|  | |
| file | FDRDistribution.java |
|  | |
| file | FisherCombinedProbabilityTest.java |
|  | |
| file | KSTest.java |
|  | |
| file | Loess.java |
|  | |
| file | MannWhitney.java |
|  | |
| file | MannWhitneyPermutations.java |
|  | |
| file | MathUtil.java |
|  | |
| file | PermutationDistribution.java |
|  | |
| file | ScanStatistics.java |
|  | |
| file | Statistics.java |
|  | |
| file | UnfairDie.java |
|  | |
| file | Wilcox2Sample.java |
|  | |


---

Generated on Fri Aug 15 2014 15:11:15 for ESAT by  

 1.8.7
